# Supplementary material for: AMPK induces degradation of the transcriptional repressor PROX1 impairing branched amino acid metabolism and tumourigenesis
Source: Nat Commun. 2022 Nov 24;13:7215. doi: 10.1038/s41467-022-34747-y (PMC9700865; doi:10.1038/s41467-022-34747-y)
Supplement: Supplementary file 1 — Supplementary Information [file 41467_2022_34747_MOESM1_ESM.pdf]

Supplementary Information

AMPK induces degradation of the transcriptional repressor PROX1 impairing branched amino acid metabolism and tumourigenesis

Yanan Wang, Mengjun Luo, Fan Wang, Yu Tong, Linfeng Li, Yu Shu, Ke Qiao, Lei Zhang, Guoquan Yan, Jing Liu, Hongbin Ji, Youhua Xie, Yonglong Zhang, Wei-Qiang Gao, Yanfeng Liu

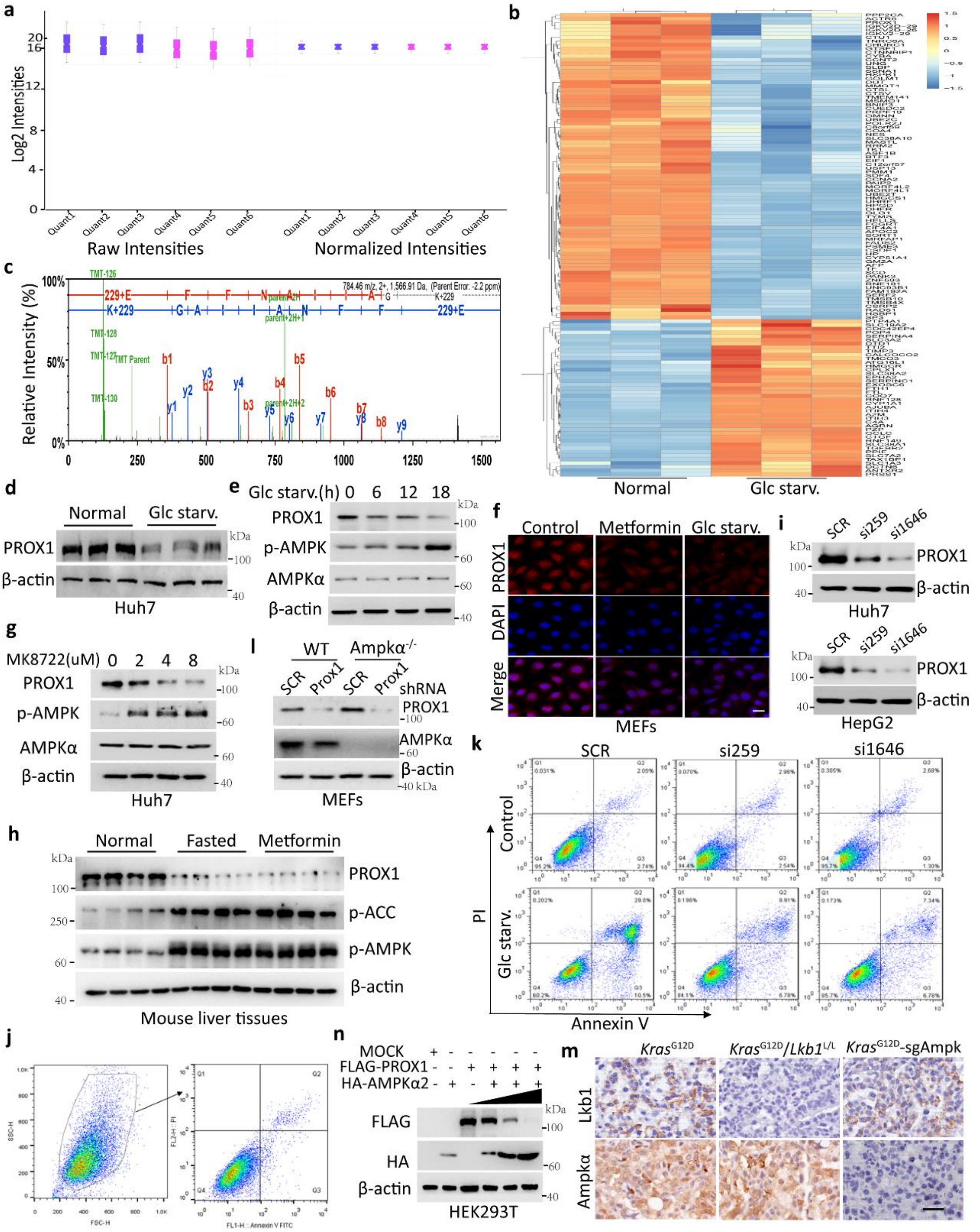

Supplementary Fig. 1 PROX1 is identified as a novel factor to response metabolic stress.

a The relative raw and normalized intensities of total proteins identified from every sample (n=6) by TMT6 labeled quantitative proteomics are shown as indicated. Raw intensities Quant1/ Quant2/ Quant3/ Quant4/ Quant5/ Quant6: n=5228/5228/5228/5228, maximum=22.9/22.7/22.9/22.5/22.3/22.5, upper quartile=16.2/16/16.2/15.7/15.515.8,

lower quartile=15.4/15.2/15.4/14.9/14.7/15, minimum=10.9/10.7/11.1/9.13/9.75/10.3.

Normalized intensities Quant1/ Quant2/ Quant3/ Quant4/ Quant5/ Quant6: n=5228/5228/5228/5228, maximum=22.6/22.6/22.6/22.7/22.6/22.6, upper quartile=15.9/15.9/15.9/15.9/15.9/15.9, lower quartile=15.1/15.1/15.1/15.1/15.1/15.1, minimum=10.6/10.6/10.8/9.35/10.1/10.4. **b** The heatmap of the differential proteins from Huh7 cells with or without glucose deprivation treatment. **c** PROX1 peptides identified through liquid chromatography-tandem mass spectrometry are shown. **d**, **e** Western blot analysis the level of PROX1 in the Huh7 cells (**d**) and MEFs (**e**) in response to glucose starvation (Glc starv.) as indicated. **f** Immunofluorescence assay shows the expression of PROX1 in the MEFs upon glucose starvation and metformin treatment (5mM) for 12h. Scale bar, 10 μm. The immunofluorescences are repeated independently with similar results at three times. **g** Western blot analysis the level of PROX1 in the Huh7 cells in response to MK8722, a selective on-target synthetic AMPK activator. **h** Liver tissues from normal, fasted and metformin (500mg/L) treatment mice for 24h are subjected to western blot (n=4). **i** The western blot analysis of the Huh7 and HepG2 cells were infected with the lentivirus either expressing *PROX1* siRNA (si259 or si1646) precursor or scrambled siRNA precursor (SCR). **j** Gating strategy to determine the percentage of apoptotic cells. **k** The apoptotic analysis of the Huh7 cells were infected with the lentivirus as the indicated by flow cytometry. **l** The western blot analysis of the wild-type (WT) and AMPKα knockout (Ampkα$^{-/-}$) MEFs were infected with the lentivirus either expressing scrambled siRNA precursor (SCR) or *Prox1* siRNA (shProx1) as the indicated. **m** Immunohistochemistry analysis of the levels of Lkb1 and Ampkα in the murine lung tumour tissues from *Kras*^{G12D}, *Kras*^{G12D}; *Lkb1*$^{flox/flox}$ (*Kras*^{G12D}/*Lkb1*$^{L/L}$) and Ampkα KO *Kras*^{G12D} (*Kras*^{G12D}-sgAmpk) mouse (n=5). Scale bar, 50 μm. **n** The western blot analysis of the cell lysates as indicated. The immunoblots are repeated independently with similar results at three times. Source data are provided as a Source data file.

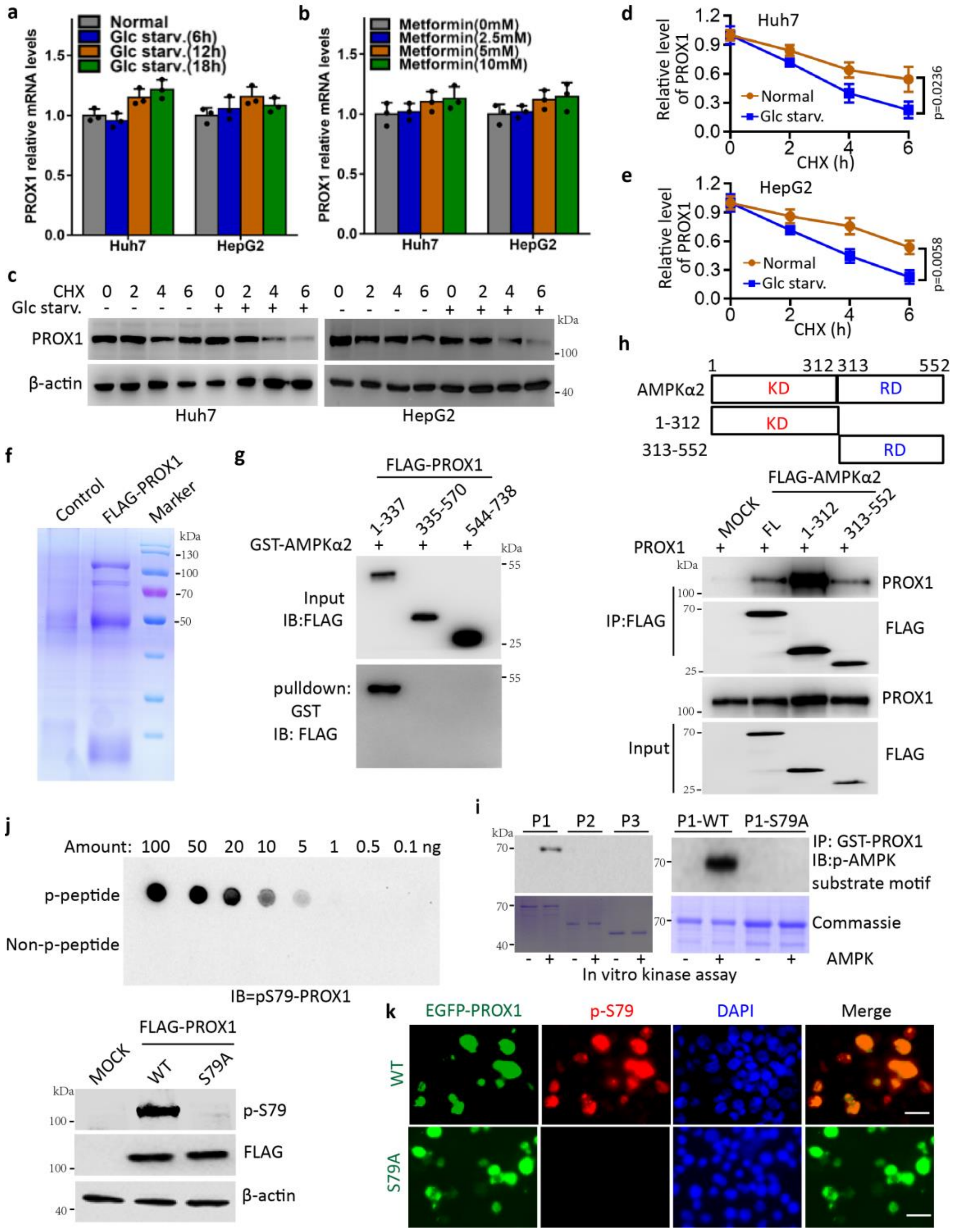

Supplementary Fig. 2 PROX1 is a novel substrate of AMPK.

a, **b** Realtime-PCR assay shows the expression of PROX1 in the Huh7 and HepG2 cells upon glucose starvation (**a**) and metformin treatment (**b**) as indicated (n=3 independent experiments). **c-e** Huh7 and HepG2 cells upon glucose starvation or not were subject to cycloheximide (CHX, 100mg/mL) experiment as indicated. Representative western blot (**c**) and the corresponding quantified graph of Huh7 (**d**) and HepG2 (**e**) cells are

shown. n=3 biologically independent experiments. **f** HEK293T transfected with FLAG-PROX1 or control plasmid were subject to Co-IP/MS. The assay is repeated independently with similar results at two times. **g** HEK293T cells expressing FLAG-PROX1 fragment incubated with purified GST-AMPKα2. The interaction was detected by anti-FLAG mAb after GST-pulldown as indicted. **h** Mapping of the PROX1-interacting regions of AMPKα2. Upper, the domain organization of AMPKα2 and the deletion constructs. KD, kinase domain; RD, regulatory domain; Lower, co-IP was performed with an anti-FLAG mAb and captured PROX1 was detected by an anti-PROX1 polyclonal antibody as indicated. **i** Each GST-PROX1 fragment (left), purified recombinant PROX1 fragment (P1-WT) and replacement of S79 with Ala (A) in the PROX1 (P1-S79A) incubated with or without purified AMPK. p-AMPK substrate was detected by anti-phospho-AMPK substrate motif mAb after GST-pulldown. **j** Specificity of phosphor-S79-PROX1 antibody was determined by dot blot assay (upper). PVDF membrane was spotted with indicated amounts of phosphor-S79 peptide or un-phosphorylated peptide, probed with pS79-PROX1 antibody. Immunoblot analysis of the cell lysates from HEK293T cells expressing FLAG-PROX1 variants with the indicated antibodies (down). **k** Immunofluorescence analysis of the Huh7 cells expressing EGFP-PROX1 variants with the indicated antibodies (n=3 independent experiments). Scale bar, 25 μm. IB, immunoblot; IP, immunoprecipitation. Relative ratio of individual protein abundance was determined from signal intensity. The immunoblots are repeated independently with similar results at least three times. For **a, b, d** and **e**, data represent the mean ± SD. Statistical significance was assessed using two-tailed unpaired Student's t-test. Source data are provided as a Source Data file.

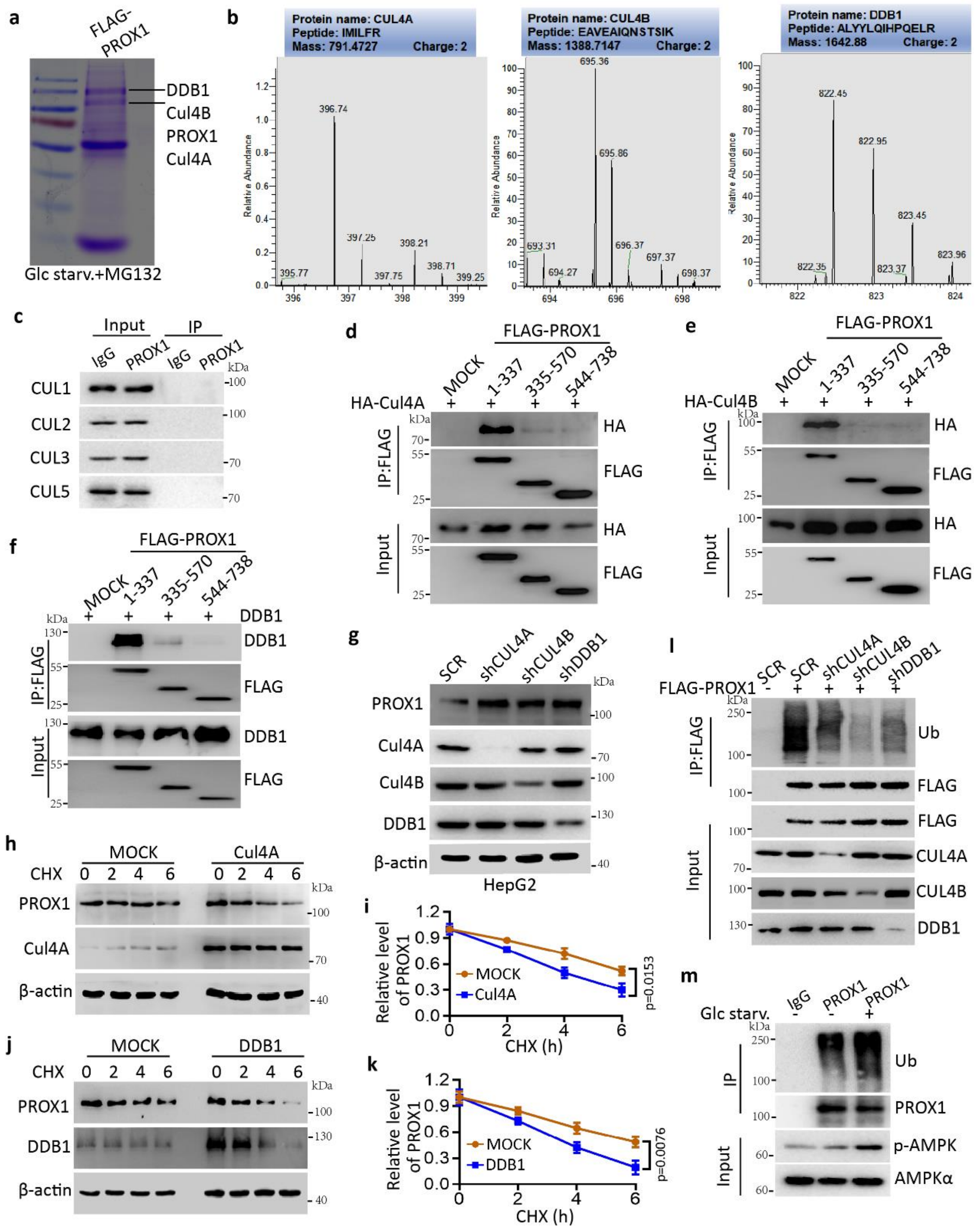

Supplementary Fig. 3 PROX1 is a novel substrate of CUL4-DDB1 E3 ligase complex, which directly reduces PROX1 protein stability.

a Co-IP/MS was conducted to identify E3 ligase associated with PROX1 upon glucose starvation and MG132 treatment (4uM) for 8h. **b** The peptides of CUL4A (left), CUL4B (middle), and DDB1 (right) identified by Co-IP/MS are shown as the indicated. **c** Endogenous PROX1 in the Huh7 cells treated with MG132 (4uM) was immunoprecipitated using anti-PROX1 antibody or isotype IgG control and subjected

to immunoblot analysis. **d-f** HEK293T cells were co-transfected with FLAG-PROX1 fragment and HA-CUL4A (**d**), and HA-CUL4B (**e**), or DDB1 (**f**) plasmids. Immunoblot analysis of the cell lysates and FLAG-IP with the indicated antibodies. **g** Immunoblot analysis of the cell lysates from HepG2 cells with the indicated antibodies. **h**, **i** Huh7 cells infected with Control and CUL4A lentivirus were subject to cycloheximide (CHX, 100mg/mL) experiment as indicated. n=3 biologically independent experiments. Representative western blot (**h**) and the corresponding quantified graph (**i**) are shown. **j**, **k** Huh7 cells infected with Control and DDB1 lentivirus were subject to cycloheximide (CHX, 100mg/mL) experiment as indicated. n=3 biologically independent experiments. Representative western blot (**j**) and the corresponding quantified graph (**k**) are shown. **l** Ubiquitination levels of PROX1 following knockdown of CUL4A, 4B and DDB1 as the indicated. **m** Ubiquitination levels of PROX1 in the Huh7 cells treated with MG132 (4uM) with or without glucose starvation were immunoprecipitated using anti-PROX1 antibody or isotype IgG control and subjected to immunoblot analysis. IB, immunoblot; IP, immunoprecipitation. The immunoblots are repeated independently with similar results at least three times. For **i** and **k**, data represent the mean ± SD. Statistical significance was assessed using two-tailed unpaired Student's t-test. Source data are provided as a Source Data file.

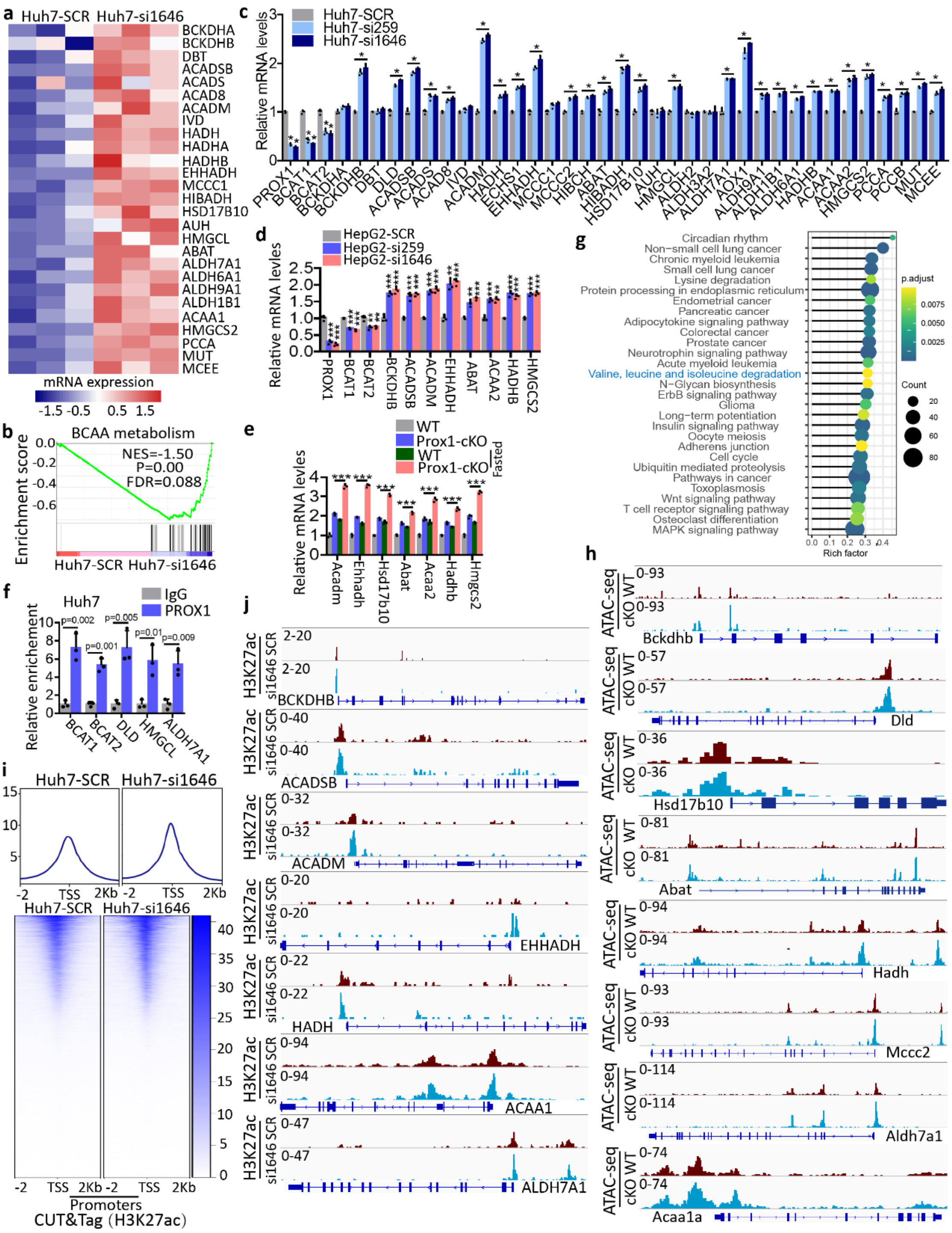

Supplementary Fig. 4 PROX1 inhibits BCAA degradation through mediating epigenetic modification.

a Heatmap demonstration of the gene expression related to valine, leucine and isoleucine degradation (BCAA metabolism) in the RNA-seq data from Huh7 cells infected with the lentivirus expressing *PROX1* siRNA (si1646) or scrambled siRNA precursor (SCR). **b** GSEA shows the enrichment of BCAA metabolism in the PROX1 knockdown Huh7 cells. Statistical significance was assessed using Permutation test. **c,**

d Realtime-PCR analysis the relative mRNA levels of BCAA metabolism genes from Huh7 (**c**) and HepG2 (**d**) cells infected with the lentivirus either expressing *PROX1* siRNA (si259 or si1646) precursor or scrambled siRNA precursor (SCR) as indicated (n=3 independent experiments). **e** Realtime-PCR analysis the relative mRNA levels of BCAA metabolism genes from $Prox1^{f/f}$ (WT) and Alb-Cre; $Prox1^{f/f}$ (Prox1-cKO) mice treated as indicated (n=3). **f** ChIP-qrtPCR was performed with sonicated chromatins immunoprecipitated from Huh7 cells by anti-PROX1 mAb or pre immune IgG (control) (n=3 independent experiments). **g** KEGG pathway enrichment analysis the ATAC-seq results from WT and Prox1-cKO mice using the differentially opened at the promoters of genes (log2Fold change>1 or <-1 and padj<0.001). Statistical significance was assessed using hypergeometric test. **h** Density maps for ATAC-seq at BCAA metabolism genes from WT and Prox1-cKO mice. **i** Heatmap of the gene peaks by Chip-seq from Huh7 cells as indicated with H3K27ac antibody. **j** Density maps for H3K27ac at BCAA metabolism genes. For **c, d, e** and **f**, data represent the mean ± SD and *P<0.05, **P<0.01, ***P<0.001. Statistical significance was assessed using two-tailed unpaired Student's t-test. Source data are provided as a Source Data file.

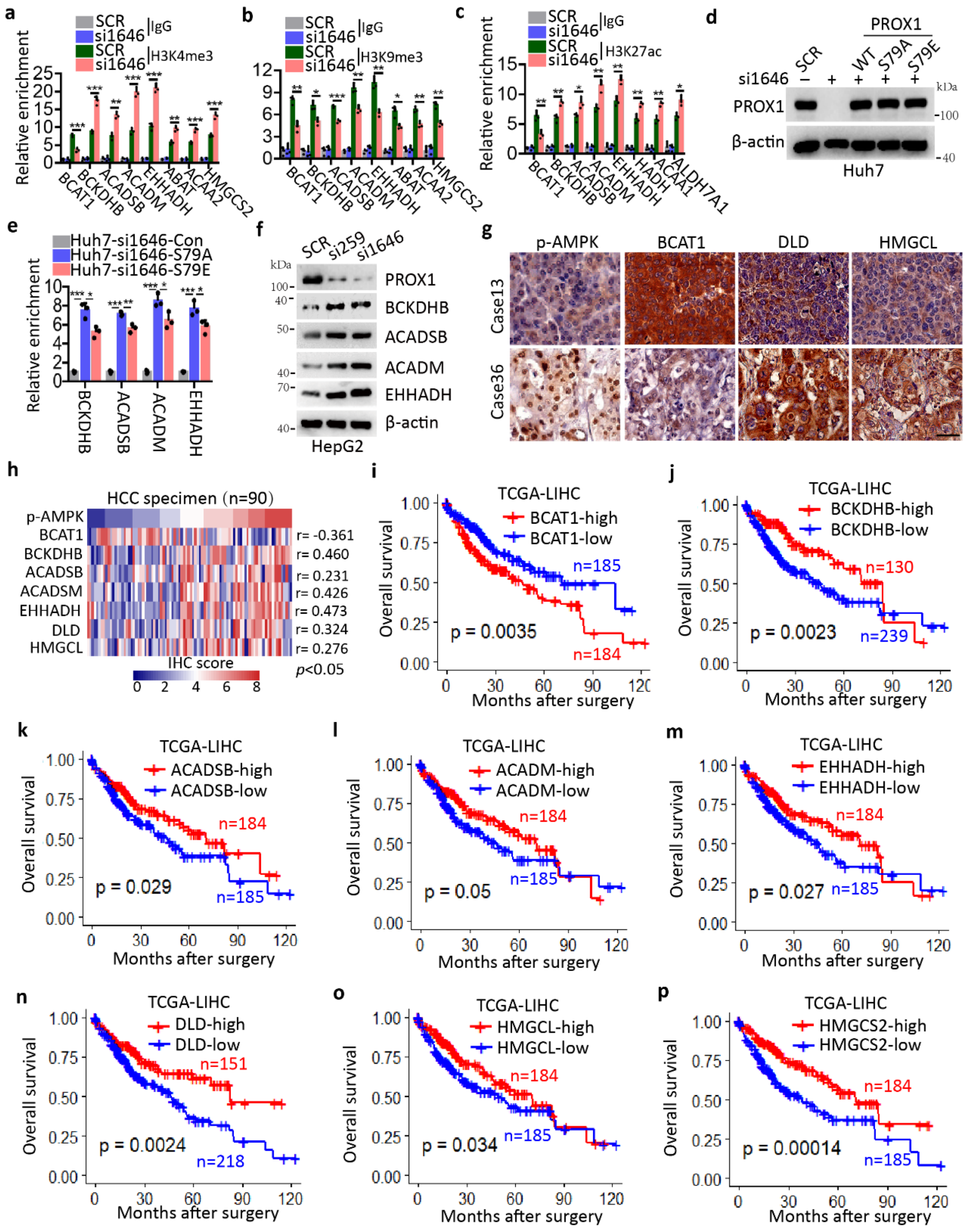

Supplementary Fig. 5 The key enzyme levels of BCAA metabolism in HCC are important for patient clinical outcomes.

a-c ChIP analysis of H3K4me3 (**a**), H3K9me3 (**b**) and H3K27ac (**c**) enrichment at the BCAA embolism genes promoter in the Huh7 cells as indicated (n=3 independent experiments). **d** The western blot analysis of the Huh7 cells infected with siRNA targeting PROX1 (si1646) re-expressing wildtype PROX1 or its S79A/S79E mutants

as indicated. **e** ChIP-qrtPCR was performed with sonicated chromatins immunoprecipitated from Huh7 cells infected with siRNA targeting PROX1 (si1646) re-expressing PROX1 S79A/S79E mutants as indicated. n=3 biologically independent experiments. **f** Immunoblot analysis of the cell lysates from the HepG2 cells infected with the lentivirus either expressing *PROX1* siRNA (si259 or si1646) precursor or scrambled siRNA precursor (SCR) as indicated. **g, h** Representative IHC staining images and the heatmap of IHC score (by Pearson's) between p-AMPK and several proteins as indicated in HCC tissues (n=90). Scale bar, 50 μm. **i-p** Kaplan–Meier analysis of overall survival probability of BCAT1, BCKDHB, ACADSB, ACADM, EHHADH, DLD, HMGCL and HMGCS2 levels in the liver hepatocellular carcinoma (LIHC) patients from TCGA database. The statistical significance was assessed using two-sided log-rank test, log-rank *p* values were shown. The immunoblots are repeated independently with similar results at least three times. For **a, b, c** and **e**, data represent the mean ± SD and *P<0.05, **P<0.01, ***P<0.001. Statistical significance was assessed using two-tailed unpaired Student's t-test. Source data are provided as a Source Data file.

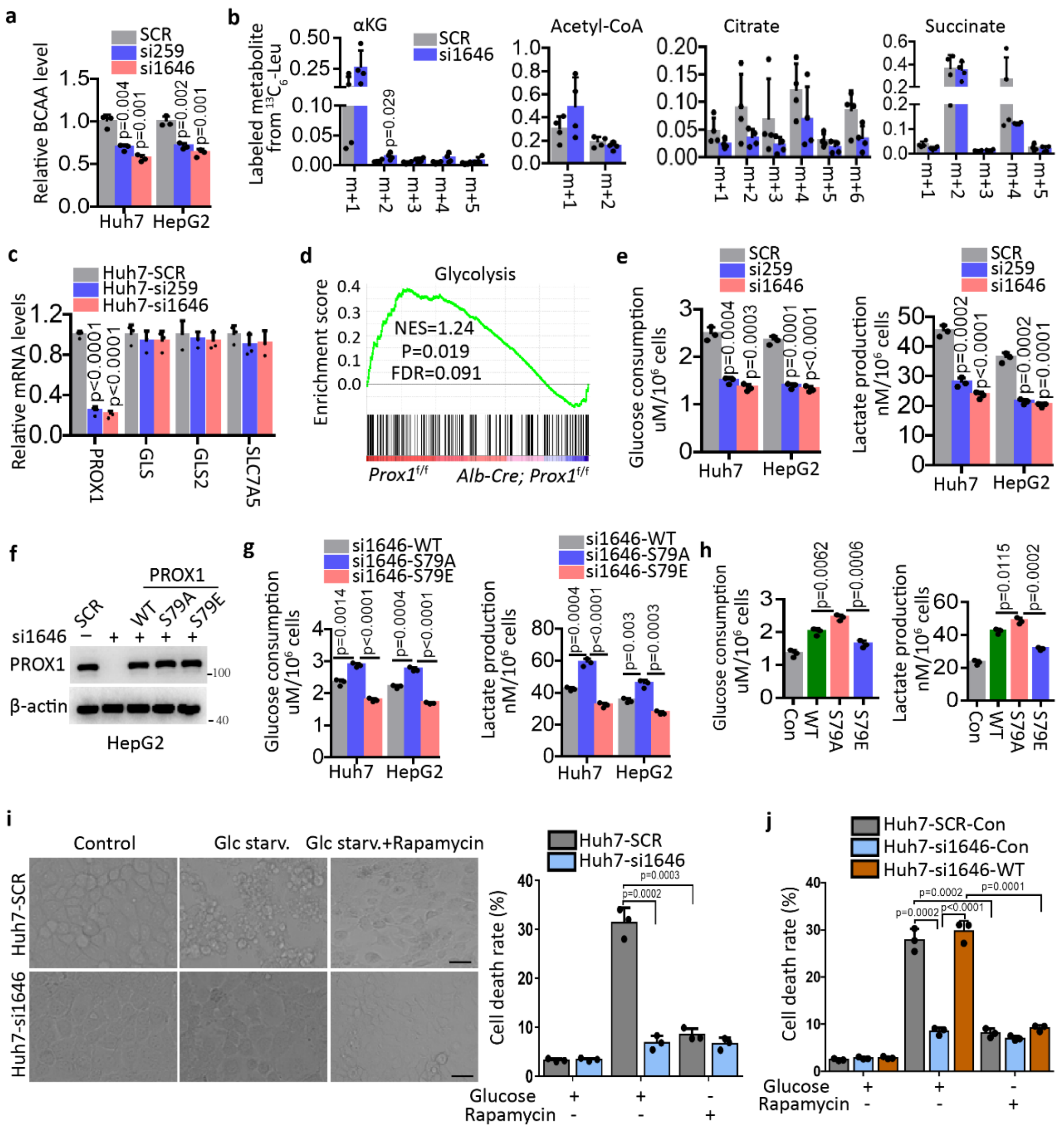

Supplementary Fig. 6 PROX1 Ser79 phosphorylation results in its altered activity to rewire glucose metabolism.

a Relative abundance of BCAA in the HCC cells infected with the lentivirus either expressing *PROX1* siRNA (si259 or si1646) precursor or scrambled siRNA precursor (SCR) as indicated (n=3 independent experiments). **b** LC/MS analysis of the labeled metabolites in the Huh7 cells infected siRNA targeting PROX1 (si1646) and scrambled siRNA (SCR) were traced 24h with [^{13}C]-Leu-M+6 (n=4 biological replicates). The relative labeled metabolites were calculated using the ration of the counts of labeled and the total of each metabolite. **c** Realtime-PCR analysis the relative mRNA levels of GLS, GLS2 and SLC7A5 from Huh7 cells as indicated (n=3 independent experiments).

d GSEA shows the enrichment of Glycolysis in the *Prox1*$^{f/f}$ (WT) mice. Statistical significance was assessed using Permutation test. **e** Glucose consumption and lactate production of Huh7 and HepG2 cells infected with indicated lentivirus were determined (n=3 independent experiments). **f** The western blot analysis of the HepG2 cells infected with siRNA targeting PROX1 (si1646) re-expressing wild-type PROX1 or its S79A/S79E mutants as indicated (n=3 biologically independent experiments). **g** PROX1-depleted Huh7 and HepG2 cells were infected with WT PROX1 or its S79A/S79E mutants. Glucose consumption and lactate production were determined as indicated (n=3 independent experiments). **h** Glucose consumption and lactate production of SMCC-7721 cells overexpressing PROX1 variants were determined (n=3 independent experiments). **i** Huh7 infected with the indicated lentivirus upon glucose starvation with or without rapamycin. Bright field images (left) of the above cells are shown, and the cell death rate analysis (right) was used trypan blue staining to determine cell viability as the indicated (n=3 independent experiments). Scale bar, 25 μm. **j** The cell death rate analysis the Huh7 infected with siRNA targeting PROX1 (si1646) re-expressing wild-type PROX1 upon glucose starvation with or without rapamycin by trypan blue staining to determine cell viability as the indicated (n=3 independent experiments). Scale bar, 50 μm. For **a-c, e** and **g-j**, data represent the mean ± SD. Statistical significance was assessed using two-tailed unpaired Student's t-test. Source data are provided as a Source Data file.

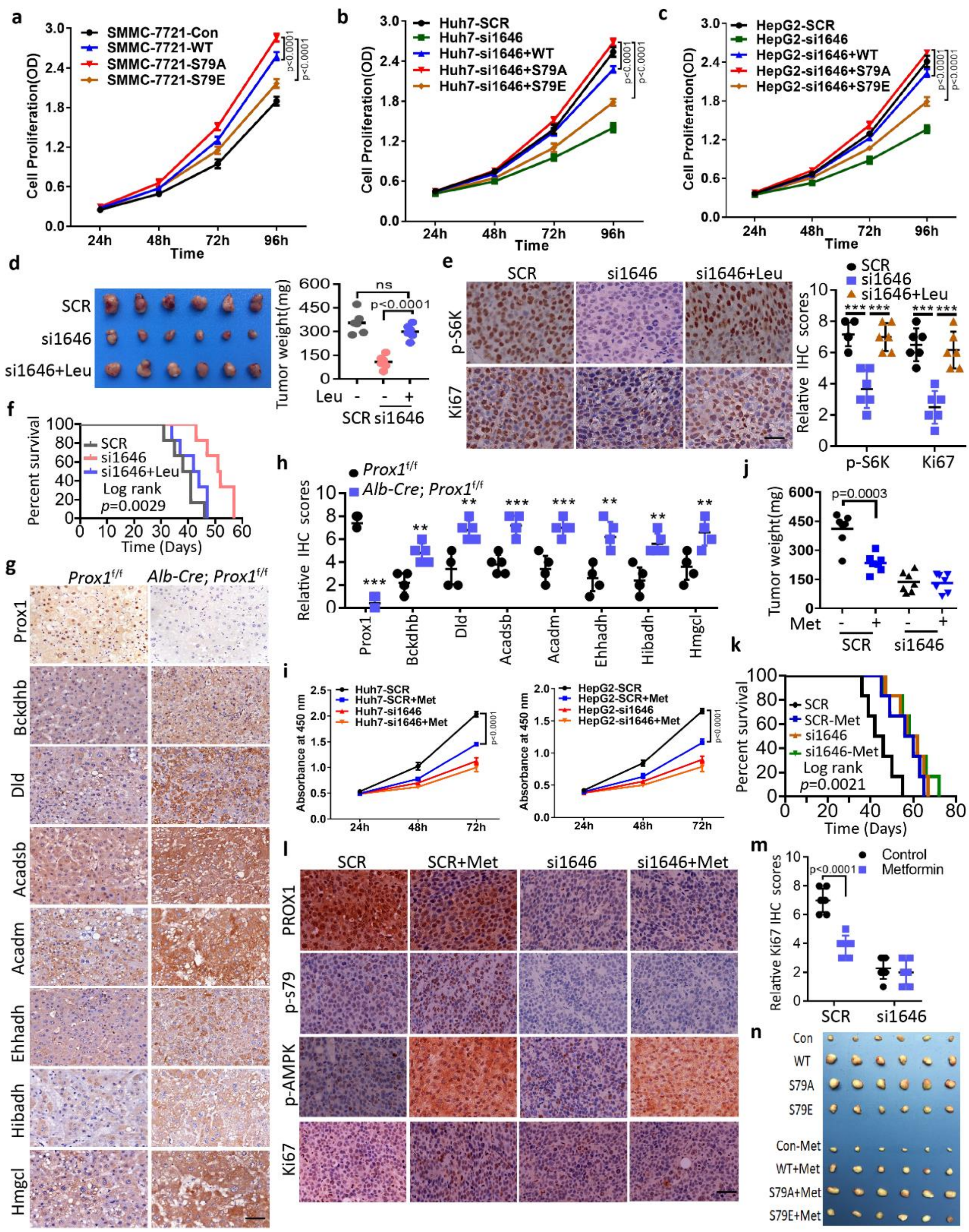

Supplementary Fig. 7 AMPK-PROX1 axis controls liver tumour progression and therapeutic response.

a-c CCK8 assays analysis the cell proliferation from SMCC-7721 (**a**), Huh7 (**b**), and HepG2 (**c**) cells infected with the indicated lentivirus (n=6 biological replicates). **d** Huh7 cells stably expressing the indicated siRNAs were subcutaneously injected into in nude mice respectively with or without Leu (1.0 g/L) treatment for 25d. Shown are

representative image and the tumour weights (n=6). **e** Representative IHC staining images and statistical data of p-S6K and Ki-67 expression in HCC tissues of mice inoculated with Huh7 cells stably expressing the indicated siRNAs were subcutaneously injected into in nude mice (n=6) respectively with or without Leu (1.0 g/L) treatment as indicated. Scale bar, 50 μm. **f** Kaplan–Meier analysis of overall survival percentage in nude mice inoculated with the above Huh7 cells. The statistical significance (p=0.0029) was assessed using log-rank test according to mice with Huh7-si1646 cells with or without Leu (1.0 g/L) treatment. **g, h** Representative IHC staining images (**g**) and statistical data (**h**) of protein expressions in the murine liver tumour tissues from *Prox1*$^{f/f}$ (WT) and Alb-Cre; *Prox1*$^{f/f}$ (Prox1-cKO) mice (n=5). Scale bar, 50 μm. **i** CCK8 assays analysis the cell proliferation from Huh7 and HepG2 cells as indicated (n=3 independent experiments). **j** Huh7 cells stably expressing the indicated siRNAs were subcutaneously injected into in nude mice respectively with or without metformin treatment (n=7). Shown are weights of tumours at day 30. **k** Kaplan–Meier analysis of overall survival percentage in nude mice as indicated. The statistical significance (p=0.0021) was assessed using log-rank test. **l**, **m** Representative IHC staining images (**l**) and statistical data (**m**) of Ki-67 expression in the above tissues as indicated (n=7). Scale bar, 50 μm. **n** SMCC-7721 cells stably overexpressing the PROX1 variants were subcutaneously injected into nude mice respectively with or without metformin (500mg/L) treatment as indicated. Shown are representative image (n=6). For **a-e, h-j** and **m**, data represent the mean ± SD and *P<0.05, **P<0.01, ***P<0.001. Statistical significance was assessed using two-tailed unpaired Student's t-test. Source data are provided as a Source Data file.

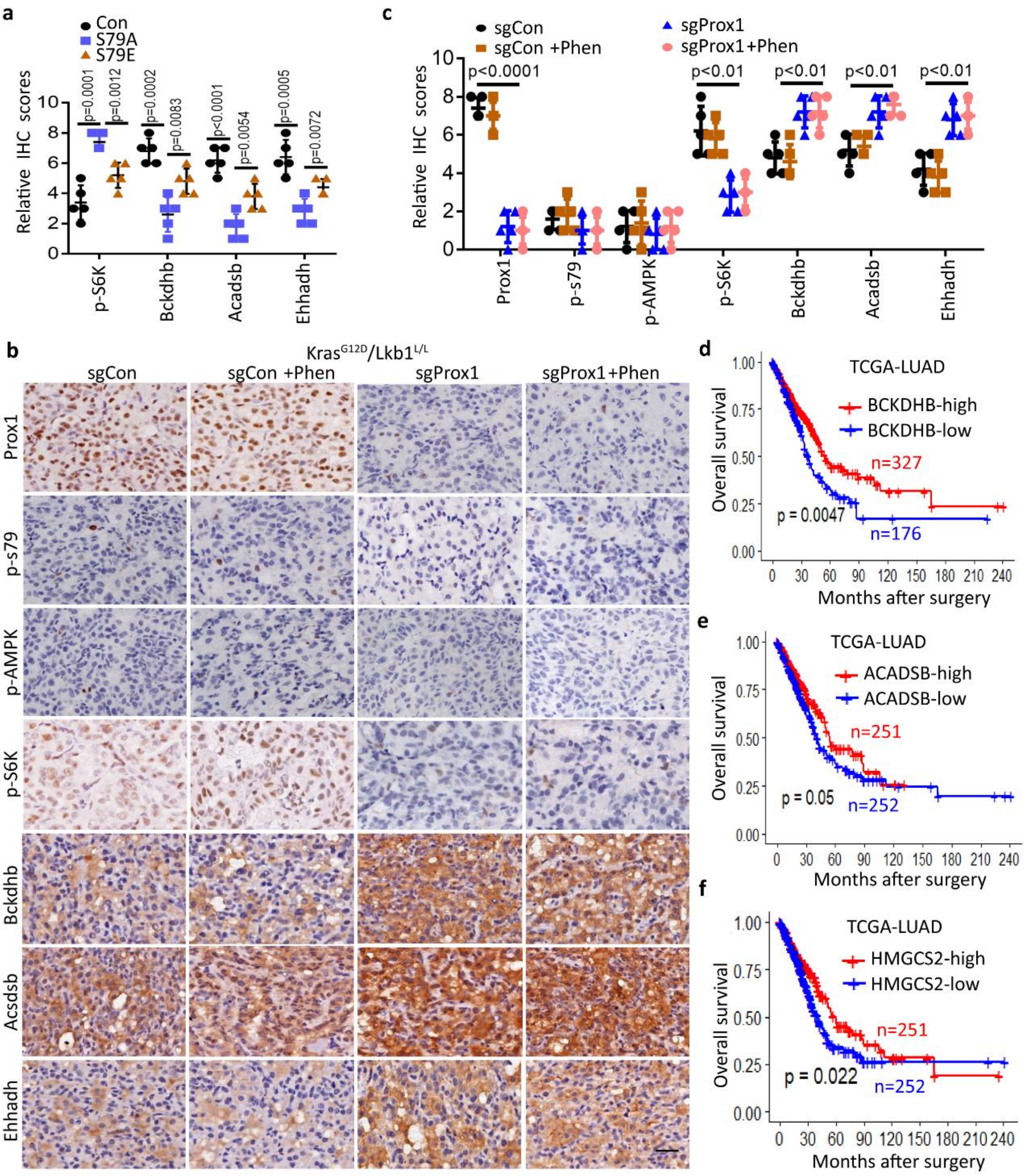

Supplementary Fig. 8 PROX1 inhibits the BCAA metabolism in the *Kras*^{G12D}/*Lkb1*$^{L/L}$ mice.

a Immunohistochemistry analysis of the relative levels of p-S6K, Bckdhb, Acadsb and Ehhadh in lungtumour tissues from *Kras*^{G12D}/*Lkb1*$^{L/L}$ mice treated with lentivirus as indicated (n=5 mice). **b, c** Representative IHC staining images (**b**) and statistical data (**c**) of Prox1, p-S79, p-AMPK, p-S6K, Bckdhb, Acadsb and Ehhadh expression in the murine lungtumour tissues from *Kras*^{G12D}/*Lkb1*$^{L/L}$ mice treated with a lenti-Cre-sgPROX1 and sgCon virus as indicated (n=5 mice) with or without phenformin (1.5g/L)

treatment. Scale bar, 50 μm. **d-f** Kaplan–Meier analysis of overall survival probability of BCKDHB, ACADSB, and HMGCS2 levels in the lung adenocarcinoma (LUAD) patients from TCGA database. The statistical significance was assessed using two-sided log-rank test, log-rank *p* values were shown. For **a** and **c**, data represent the mean ± SD. Statistical significance was assessed using two-tailed unpaired Student's t-test. Source data are provided as a Source Data file.

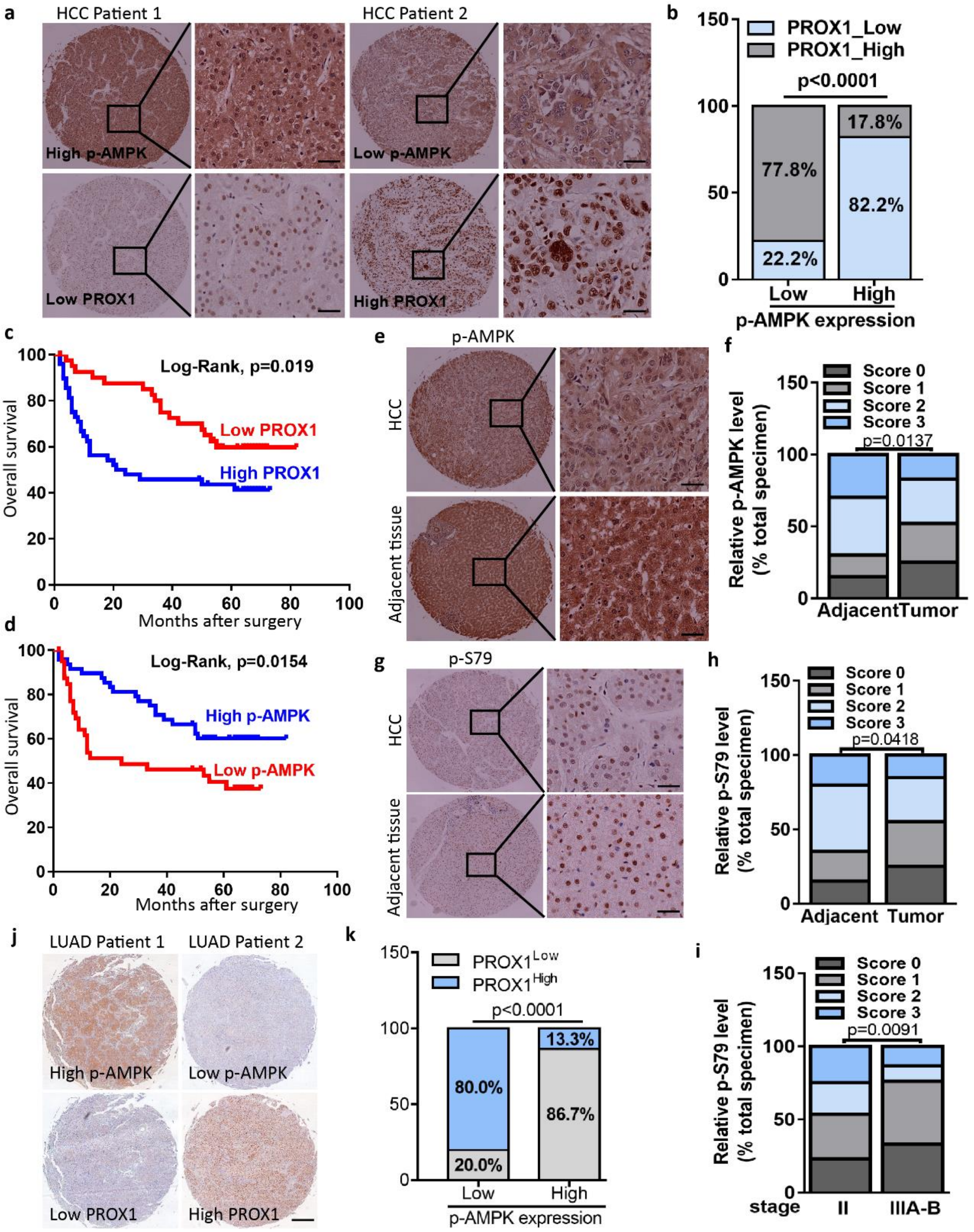

Supplementary Fig. 9 AMPK is negatively correlated with the PROX1 expression in HCC and LUAD.

a, **b** Representative IHC staining images (**a**) and statistical data (**b**) of PROX1 and p-AMPK expression in HCC tissues (n=90). Scale bar, 50 µm. **c** Kaplan–Meier analysis of overall survival probability of PROX1 levels in HCC patients. The statistical significance (p=0.019) was assessed using log-rank test according to HCC patients with

low or high expression of PROX1. **d** Kaplan–Meier analysis of overall survival probability of p-AMPK levels in HCC patients. The statistical significance (p=0.0154) was assessed using log-rank test according to HCC patients with low or high expression of p-AMPK. **e**, **f** Representative images (**e**) of IHC staining and the relative IHC scores (**f**) of p-AMPK in HCC tissues and adjacent normal tissues (n=45). Scale bar, 50 μm. **g**, **h** Representative images (**g**) of IHC staining and the relative IHC scores (**h**) of p-S79 in HCC tissues and adjacent normal tissues (n=45). Scale bar, 50 μm. **i** Representative the relative IHC scores of p-S79 in HCC tissues as indicated. **j**, **k** Representative IHC staining images (**j**) and statistical data (**k**) of PROX1 and p-AMPK expression in lung adenocarcinoma (LUAD) tissues (n=90). Scale bar, 50 μm. For **b, f, h, i** and **k**, statistical significance was assessed using two-sided Chi-square test. Source data are provided as a Source Data file.

Supplementary Table 1. Primer sequences for quantitative real-time PCR.

Hu-BCAT1-F	CAACTATGGAGAATGGTCCTAAGCT
Hu-BCAT1-R	TGTCCAGTCGCTCTCTTCTCTTC
Hu-BCAT2-F	CGCTCCTGTTCGTCATTCTCT
Hu-BCAT2-R	CCCACCTAACTTGTAGTTGCC
Hu-BCKDHA-F	CTACAAGAGCATGACACTGCTT
Hu-BCKDHA-R	CCCTCCTCACCATAGTTGGTC
Hu-BCKDHB-F	GATTTGGAATCGGAATTGCGG
Hu-BCKDHB-R	CAGAGCGATAGCGATACTTGG
Hu-DBT-F	CTGAACTGGTTAAGCTCCGAG
Hu-DBT-R	GCTCAGTATCCATTGCTATCCCA
Hu-DLD-F	GAAATGTCCGAAGTTCGCTTGA
Hu-DLD-R	TCAGCTTTCGTAGCAGTGACT
Hu-ACADSB-F	TCTTGGGACAAATTGGACATGG
Hu-ACADSB-R	GTGAGCCACTTGGTGTTGGA
Hu-ACADS-F	CGGCAGTTACACACCATCTAC
Hu-ACADS-R	GCAATGGGAAACAACTCCTTCTC
Hu-ACAD8-F	CAATTCACACTGGCTGATATGGC
Hu-ACAD8-R	ACTGCTGAACAGCGTAATCCT
Hu-HADH-F	CACACAGTAGTGTTGGTAGACC
Hu-HADH-R	TGCCACTTTCCTAAGGCTTTC
Hu-ECHS1-F	TGTCCTGTTGAGACACTGGTG
Hu-ECHS1-R	ACAAACGCGGTCATCCCTTC
Hu-EHHADH-F	TCCTGTGATTGCTGTAGACTCG
Hu-EHHADH-R	GGCCGCTCTGTTGCATTTTG
Hu-HIBCH-F	GCAATTTCGAGTGGCTACAGA
Hu-HIBCH-R	CCTTGGAGTCGTGGCAAGAA
Hu-HIBADH-F	TGCTGCCCACCAGTATCAATG
Hu-HIBADH-R	GCAGGATCAATAGTGCTGGAATC
Hu-ABAT-F	AAGAGAGCCGAGGCAATTACC
Hu-ABAT-R	GCTCGCATTTTGAGGCTGTTG
Hu-ALDH2-F	ATCCCCATTGACGGAGACTTC
Hu-ALDH2-R	CACAACCACGTTTCCAGTTGC
Hu-ALDH3A2-F	CTGAAGCAGCGATTTGACCAC
Hu-ALDH3A2-R	CCCTCCCAGTTCAAGAGTCAC
Hu-ALDH7A1-F	GATGATTGGAGGACCTATCTTGC
Hu-ALDH7A1-R	ACAGCCACACTAATGAGGGAA
Hu-AOX1-F	ATGCCTGTCTGATTCCCATCT
Hu-AOX1-R	CATGACACTTGGCAATCCTCT
Hu-ALDH9A1-F	TGGAGTCAAAAATCTGGCATGG
Hu-ALDH9A1-R	AGTAGCAATTTCATCCTCCCGT
Hu-ALDH1B1-F	AGAGTCTTACGCCTTGGACTT

Hu-ALDH1B1-R	GTCTTGCCATGCCACTTGTC
Hu-MUT-F	TGGGTTTGCCAACTGTGAAAA
Hu-MUT-R	GGTATTCCCTCAGCTACAGCTT
Hu-PCCA-F	AAGCTACCTCAACATGGATGC
Hu-PCCA-R	GTGTCAGGTCCAATGAAAACGA
Hu-MCEE-F	ATGTAGCCATAGCAGTGCCAG
Hu-MCEE-R	GACTGTCACGTCCCAATGGAT
Hu-ALDH6A1-F	GGCAGACACTTCAGTATTAAGCC
Hu-ALDH6A1-R	AGAGGCAGACGGTAGGAATAAA
Hu-ACADM-F	ACAGGGGTTCAGACTGCTATT
Hu-ACADM-R	TCCTCCGTTGGTTATCCACAT
Hu-HSD17B10-F	TGGCGGTAATAACCGGAGGA
Hu-HSD17B10-R	ACAGTTGACAGCTACATCCACA
Hu-ACAA1-F	GCGGTTCTCAAGGACGTGAAT
Hu-ACAA1-R	GTCTCCGGGATGTCACTCAGA
Hu-ACAA2-F	CTGCTCCGAGGTGTGTTTGTA
Hu-ACAA2-R	GGCAGCAAATTCAGACAAGTCA
Hu-PCCB-F	TCTGCAAAATCATGGACCAGG
Hu-PCCB-R	GGATGCCGTAACATTCCTCAG
Hu-MCCC1-F	TGATGATGCTATGCTGATCGAGA
Hu-MCCC1-R	GATGTCGCCTCTGCACACTA
Hu-MCCC2-F	GAGGTGCCTACTACCCAGTGA
Hu-MCCC2-R	GCTCCTCCCGAATCAACTAAGT
Hu-AUH-F	ATTGGCGATTATTCCTGGTGG
Hu-AUH-R	GAACGTGGCTGATTAAGCCCA
Hu-HMGCL-F	TCCACTGCCATGACACCTATG
Hu-HMGCL-R	AAGCCCTCTAGCATGTAGACC
Hu-HMGCS-F	CATTAGACCGCTGCTATTCTGTC
Hu-HMGCS-R	TTCAGCAACATCCGAGCTAGA
Hu-IVD-F	ATGGCAGAGATGGCGACTG
Hu-IVD-R	TAGCCCATTGATTGCATCGTC
mouse-MCCC1-F	TGCACACCCATCTTTTTGGCT
mouse-MCCC1-R	ACCATGAAGTATGGAACAACCC
mouse-ACAA1-F	CGCTCAGAAATTGGGCGATG
mouse-ACAA1-R	TCTCCAGGACGTGAGGCTAAA
mouse-AUH-F	AGTCAGGAGTCACTTCTGGCT
mouse-AUH-R	AGCTGGCTCTAGCGTGTGA
mouse-ACAA2-F	AGCTCTGCATGACATTGCCC
mouse-ACAA2-R	CTGCTACGAGGTGTGTTCATC
mouse-DLD—F	CCTATCACTGTCACGTCAGCC
mouse-DLD—R	GAGCTGGAGTCGTGTGTACC
mouse-HIBADH-F	ACACGTCATAGAGGATGAGTGG
mouse-HIBADH-R	GCAGCGGTGTGTTCTAGGTC

mouse-BCKDHA-F	CATTGGGCTGGATGAACTCAA
mouse-BCKDHA-R	CTCCTGTTGGGACGATCTGG
mouse-IVD-F	CTCCTCGTTTAGCCCGTTGA
mouse-IVD-R	GGACGGCGAGTTTCCAGTT
mouse-MCEE-F	TTTTCCAAATCTGGCACTGCTA
mouse-MCEE-R	ATGAGGCGTGTAGTGAAGGC
mouse-ALDH6A1-F	TCCCTTGTTCCAGTGTGATTAAC
mouse-ALDH6A1-R	AGCCGTTGAGTCCTGCAAAC
mouse-HMGCS2-F	GTCCACATATTGGGCTGGAAA
mouse-HMGCS2-R	GAAGAGAGCGATGCAGGAAAC
mouse-ALDH1B1-F	CATGCCACTCGTTGTTGATGA
mouse-ALDH1B1-R	CTCCAGGGCAGGACTACCTC
mouse-PCCB-F	TGCGCTCTTTAACTGAAACCG
mouse-PCCB-R	GCGGCGATTAGGATTCGGG
mouse-HSD17B10-F	TGGGGCAAATATGCAGCTTTC
mouse-HSD17B10-R	GCTTGGTCGCGGTAGTAACTG
mouse-ALDH9A1-F	AGGCCACTTTTCTTACTCCAGA
mouse-ALDH9A1-R	GGCCGAGTGATTGCCACTT
mouse-MUT-F	CCTCTGGGTTTTTGCCTTTCAG
mouse-MUT-R	TTTTTGCTATCGCCCCATTACC
mouse-HADH-F	TGGATTTTGCCAGGATGTCTTC
mouse-HADH-R	TCAAGCATGTGACCGTCATCG
mouse-EHHADH-F	GGTCCAAACTAGCTTTCTGGAG
mouse-EHHADH-R	ATGGCTGAGTATCTGAGGCTG
mouse-HADHA-F	GTTGGCCCAGATTTCGTTCA
mouse-HADHA-R	TGCATTTGCCGCAGCTTTAC
mouse-DBT-F	GAGTGACGTGGCTGACTGTA
mouse-DBT-R	AGACTGACCTGTGTTCGCTAT
mouse-MCCC2-F	CACTCCCTCCTAGTCTCACATAC
mouse-MCCC2-R	GCCTATCACGGGGACTCAGT
mouse-HMGCL-F	GGAGCCCTGCTTCGGAAAC
mouse-HMGCL-R	CAGGTGAAGATCGTGGAAGTC
mouse-PCCA-F	GACAGCCTCATCCGCCATTTT
mouse-PCCA-R	TTCATACCAATGCCTAGTGGTGT
mouse-ACAD8-F	TCCCGAGCTGCAAAGTCAAAG
mouse-ACAD8-R	TCCTTGGGGCTAAATGAAGAAC
mouse-ECHS1-F	TGCTCGGGTGAGTCTCTGAG
mouse-ECHS1-R	TTGTGAACTTGCCATGATGTGT
mouse-ACADM-F	CCCCGCTTTTGTCATATTCCG
mouse-ACADM-R	AGGGTTTAGTTTTGAGTTGACGG
mouse-HIBCH-F	AGGAATGTGTCAGGGTCTTGT
mouse-HIBCH-R	GTGGAGGCGTCATAACGCTC
mouse-ALDH2-F	CGCCAATCGGTACAACAGC

mouse-ALDH2-R	GACGCCGTCAGCAGGAAAA
mouse-ACADSB-F	ATCCCTGGATCACCGATTTCT
mouse-ACADSB-R	CCCAACCTGCTTGTCTCCTTG
mouse-BCKDHB-F	GATTTCCGCAATAGCTGTAGCA
mouse-BCKDHB-R	CTTCCGATGCACTGTTGGTTT
mouse-AOX1-F	TGGTGACTGCTGTACCATGTAG
mouse-AOX1-R	GAGGAAGAATCTCCGACTCACA
mouse-ABAT-F	GGTTGTAACCTATGGGCACAG
mouse-ABAT-R	CTGAACACAATCCAGAATGCAGA
mouse-ALDH3A2-F	CATTGAGTTCACTTTTGCTCAGG
mouse-ALDH3A2-R	GCGGAGGATGGTGCAAGAG
