# Supplementary material for: AMPK induces degradation of the transcriptional repressor PROX1 impairing branched amino acid metabolism and tumourigenesis
Source: Nat Commun. 2022 Nov 24;13:7215. doi: 10.1038/s41467-022-34747-y (PMC9700865; doi:10.1038/s41467-022-34747-y)
Supplement: Supplementary file 3 — Reporting Summary [file 41467_2022_34747_MOESM3_ESM.pdf]

nature portfolio

Corresponding author(s): Yanfeng Liu; Wei-Qiang Gao; Yonglong Zhang; Youhua Xie

Last updated by author(s): Jul 7, 2022

Reporting Summary

Nature Portfolio wishes to improve the reproducibility of the work that we publish. This form provides structure for consistency and transparency in reporting. For further information on Nature Portfolio policies, see our Editorial Policies and the Editorial Policy Checklist.

Statistics

For all statistical analyses, confirm that the following items are present in the figure legend, table legend, main text, or Methods section.

n/a	Confirmed	
☐	☒	The exact sample size (*n*) for each experimental group/condition, given as a discrete number and unit of measurement
☐	☒	A statement on whether measurements were taken from distinct samples or whether the same sample was measured repeatedly
☐	☒	The statistical test(s) used AND whether they are one- or two-sided<br>*Only common tests should be described solely by name; describe more complex techniques in the Methods section.*
☐	☒	A description of all covariates tested
☐	☒	A description of any assumptions or corrections, such as tests of normality and adjustment for multiple comparisons
☐	☒	A full description of the statistical parameters including central tendency (e.g. means) or other basic estimates (e.g. regression coefficient) AND variation (e.g. standard deviation) or associated estimates of uncertainty (e.g. confidence intervals)
☐	☒	For null hypothesis testing, the test statistic (e.g. *F*, *t*, *r*) with confidence intervals, effect sizes, degrees of freedom and *P* value noted<br>*Give P values as exact values whenever suitable.*
☒	☐	For Bayesian analysis, information on the choice of priors and Markov chain Monte Carlo settings
☒	☐	For hierarchical and complex designs, identification of the appropriate level for tests and full reporting of outcomes
☐	☒	Estimates of effect sizes (e.g. Cohen's *d*, Pearson's *r*), indicating how they were calculated

Our web collection on statistics for biologists contains articles on many of the points above.

Software and code

Policy information about availability of computer code

Data collection	Illumina NovaSeq 6000 was used for raw data collection of RNA-seq and ATAC-seq.Thermo Scientific Xcalibur (version.3.0.63, Thermo Fisher Scientific. USA) was used for raw data collection of proteomic data. Thermo Scientific Proteome Discoverer (version.1.4.12) was used for database searching of proteomic data.
Data analysis	Image Lab software (version:3.0), Image J software (version:1.6), Flow Jo (version:7.6.1), Photoshop (version:CS5), SPSS software 20, Microsoft Excel 2017, Graphpad Prism 7, Gene Set Enrichment Analysis (GSEA, version:3.0), R version 3.6.3. Scaffold Q+ (version.4.0.5, Proteome Software, Inc., Portland, USA).

For manuscripts utilizing custom algorithms or software that are central to the research but not yet described in published literature, software must be made available to editors and reviewers. We strongly encourage code deposition in a community repository (e.g. GitHub). See the Nature Portfolio guidelines for submitting code & software for further information.

Data

Policy information about availability of data

All manuscripts must include a data availability statement. This statement should provide the following information, where applicable:

- Accession codes, unique identifiers, or web links for publicly available datasets
- A description of any restrictions on data availability
- For clinical datasets or third party data, please ensure that the statement adheres to our policy

The data of RNA-seq, ATAC-seq and proteomic datasets generated in this study can be viewed in NODE (https://www.biosino.org/node) by pasting the accession (OEP003076) into the search box. The TCGA data can been obtained from the TCGA datebase (https://xenabrowser.net/datapages/?hub=https://tcga.xenahubs.net:443). Unprocessed gel blot of Figures are provided in Source date file. Source data are provided with this paper. We have provided a full data

availability statement in the manuscript.

Field-specific reporting

Please select the one below that is the best fit for your research. If you are not sure, read the appropriate sections before making your selection.

☒ Life sciences ☐ Behavioural & social sciences ☐ Ecological, evolutionary & environmental sciences

For a reference copy of the document with all sections, see nature.com/documents/nr-reporting-summary-flat.pdf

Life sciences study design

All studies must disclose on these points even when the disclosure is negative.

Sample size	Sample size for each experiment are provided in figure legends. We used at least 5 mice per group for animal experiment, the sample size was different in each experiment (5-7) , the exact number of mice was indicated in the figure legends. No statistical method was used to determine the sample size.
Data exclusions	No data were excluded from analysis.
Replication	Experiments in the article are reliably produced, replication were described in the figure legends.
Randomization	Animals were randomly allocated to experiment groups. For in vitro experiments, the samples were prepared and treated in random order.
Blinding	Investigators were blinded to group allocation during data collection and/or analysis.

Reporting for specific materials, systems and methods

We require information from authors about some types of materials, experimental systems and methods used in many studies. Here, indicate whether each material, system or method listed is relevant to your study. If you are not sure if a list item applies to your research, read the appropriate section before selecting a response.

Materials & experimental systems

n/a	Involved in the study	
☐	☒	Antibodies
☐	☒	Eukaryotic cell lines
☒	☐	Palaeontology and archaeology
☐	☒	Animals and other organisms
☐	☒	Human research participants
☒	☐	Clinical data
☒	☐	Dual use research of concern

Methods

n/a	Involved in the study	
☒	☐	ChIP-seq
☐	☒	Flow cytometry
☒	☐	MRI-based neuroimaging

Antibodies

Antibodies used	For WB, From Cell Signaling Technology: p-AMPK (#2535, 1:1000), p-AMPK substrate motif (#5759, 1:1000), AMPKa (#2532, 1:2000), AMPKa2 (#2757, 1:2000), p-mTOR (#5536, 1:1000), mTOR (#2983, 1:2000), p-4EBP1 (#2855, 1:1000), p-S6K (#9205, 1:1000) and S6K (#9202, 1:2000); From Upstate: PROX1 (07-537, 1:2000); From Abcam: AMPKa1 (ab32047, 1:2000) and DDB1 (ab109027, 1:2000); From Sigma: Flag M2 (F3165, 1:5000) and β-Actin (A1978, 1:10000); from Thermo Fisher Scientific: HA (SG77) (71-5500, 1:3000); From Abclonal: p-S79 (customization, 1:1000), ACADM (A4567, 1:2000) and EHHADH (A13488, 1:2000); From Proteintech: CUL4A (14851-1-AP, 1:2000), CUL4B (12916-1-AP, 1:2000), BCKDHB (13685-1-AP, 1:2000) and ACADSB (13122-1-AP, 1:2000). From Cell Signaling Technology: anti-rabbit IgG, HRP-linked Antibody (#7074, 1:5000) and anti-mouse IgG, HRP-linked Antibody (#7076, 1:5000);<br>For IHC, From Cell Signaling Technology: p-AMPK (#2535, 1:200). From Abcam: DDB1 (ab109027, 1:200). From Abclonal: p-S79 (customization, 1:100), ACADM (A4567, 1:200), EHHADH (A13488, 1:200) and HIBADH (A19871, 1:200). From Proteintech: PROX1 (11067-1-AP, 1:200), CUL4A (14851-1-AP, 1:200), CUL4B (12916-1-AP, 1:200), BCAT1 (13640-1-AP, 1:200), BCKDHB (13685-1-AP, 1:200), ACADSB (13122-1-AP, 1:200), DLD (16431-1-AP, 1:200) and HMGCL (16898-1-AP, 1:200).
Validation	p-AMPK antibody, human and mouse, WB, IP and IHC, product citations:1964 (https://www.cellsignal.cn/products/primary-antibodies/phospho-ampka-thr172-40h9-rabbit-mab/2535); p-AMPK substrate motif antibody, human and mouse, WB and IP, product citations:31 (https://www.cellsignal.cn/products/primary-antibodies/phospho-ampk-substrate-motif-lxrxx-ps-pt-multimab-rabbit-mab-mix/5759); AMPKa antibody, human and mouse, WB and IP, product citations:1396 (https://www.cellsignal.cn/products/primary-antibodies/ampka-antibody/2532); AMPKa2 antibody, human and mouse, WB and IP, product citations:55 (https://www.cellsignal.cn/products/primary-antibodies/ampka2-antibody/2757); p-mTOR antibody, human and mouse, WB, IP and IF, product citations:1023 (https://www.cellsignal.cn/products/primary-antibodies/phospho-mtor-ser2448-d9c2-xp-rabbit-mab/5536);

mTOR antibody, human and mouse, WB, IHC and IF, product citations:1424 (https://www.cellsignal.cn/products/primary-antibodies/mtor-7c10-rabbit-mab/2983); p-4EBP1 antibody, human and mouse, WB, IHC and IF, product citations:1019 (https://www.cellsignal.cn/products/primary-antibodies/phospho-4e-bp1-thr37-46-236b4-rabbit-mab/2855); p-S6K antibody, human and mouse, WB, product citations:1177 (https://www.cellsignal.cn/products/primary-antibodies/phospho-p70-s6-kinase-thr389-antibody/9205); S6K antibody, human and mouse, WB and IP, product citations:1245 (https://www.cellsignal.cn/products/primary-antibodies/p70-s6-kinase-antibody/9202); PROX1 antibody, human and mouse, WB, product citations:13 (https://www.sigmaaldrich.com/CN/zh/product/mm/07537); PROX1 antibody, human and mouse, WB, FC, Chip, IHC and IF, product citations:20 (https://www.ptgcn.com/products/PROX1-Antibody-11067-2-AP); AMPKa1 antibody, human and mouse, WB, IHC and IF, product citations:95 (https://www.abcam.cn/ampk-alpha-1-antibody-y365-ab32047); DDB1 antibody, human and mouse, WB, IHC and IF, product citations:14 (https://www.abcam.cn/ddb1-antibody-epr6089-ab109027); Flag M2 antibody, all, WB, IP, IHC, IF and F, product citations:3325 (https://www.sigmaaldrich.com/catalog/product/sigma/f3165?lang=zh&region=CN); β-Actin antibody, human and mouse, WB, and IHC, product citations:3108 (https://www.sigmaaldrich.cn/CN/zh/search/a1978); HA antibody? all, WB, ICC and IF, product citations:77 (https://www.thermofisher.cn/cn/zh/antibody/product/HA-Tag-Antibody-clone-SG77);
The phospho-specific p-S79 PROX1 antibody was custom-made by abclonal, and the antibody were tested on negative controls and/or positive controls. CUL4A antibody, human and mouse, WB, IHC and IF, product citations:13 (https://www.ptgcn.com/products/CUL4A-Specific-Antibody-14851-1-AP); CUL4B antibody, human and mouse, WB, IHC and IF, product citations:47 (https://www.ptgcn.com/products/CUL4B-Antibody-12916-1-AP); ACADM antibody, human and mouse, WB, IHC and IF, (https://abclonal.com.cn/catalog/A4567); EHHADH antibody, human and mouse, WB, IHC and IF, (https://abclonal.com.cn/catalog/A13488); HIBADH antibody, human and mouse, WB and IF, KO validated (https://abclonal.com.cn/catalog/A19871); BCAT1 antibody, human and mouse, WB, IHC and IF, product citations:8 (https://www.ptgcn.com/products/BCAT1-Antibody-13640-1-AP); BCKDHB antibody, human and mouse, WB and IHC, product citations:1 (https://www.ptgcn.com/products/BCKDHB-Antibody-13685-1-AP); ACADSB antibody, human and mouse, WB, IHC and IF, product citations:1 (https://www.ptgcn.com/products/ACADSB-Antibody-13122-1-AP); DLD antibody, human and mouse, WB, IHC and IF, product citations:4 (https://www.ptgcn.com/products/DLD-Antibody-16431-1-AP); HMGCL antibody, human and mouse, WB, IHC and IF, product citations:2 (https://www.ptgcn.com/products/HMGCL-Antibody-16898-1-AP); Anti-rabbit IgG, HRP-linked antibody, human and mouse, WB, IP, IHC and ELISA, product citations:4146 (https://www.cst-c.com.cn/products/secondary-antibodies/anti-rabbit-igg-hrp-linked-antibody/7074); Anti-mouse IgG, HRP-linked antibody, human and mouse, WB, IP, IHC and ELISA, product citations:2216 (https://www.cst-c.com.cn/products/secondary-antibodies/anti-mouse-igg-hrp-linked-antibody/7076);p-S79 antibody, human and mouse, WB, IF and IHC (Supplementary Fig 1j,1k, 7i and Fig 2h, 2j, 2k, 2l, 2m, 2o);

Eukaryotic cell lines

Policy information about cell lines

Cell line source(s)	Human cell lines Huh7, HepG2, SMMC-7721 and embryo kidney cell line HEK293T were obtained from Cell Bank of Shanghai Institutes of Biological Sciences, Chinese Academy of Sciences (Shanghai, China).
Authentication	Huh7, HepG2 and HEK293T have been authenticated by STR profiling. SMMC-7721 is not authenticated. All cell lines were kept at low passages in order to maintain their identity.
Mycoplasma contamination	All cell lines were tested to be mycoplasma negative.
Commonly misidentified lines (See ICLAC register)	None of commonly misidentified cell lines were used.

Animals and other organisms

Policy information about studies involving animals; ARRIVE guidelines recommended for reporting animal research

Laboratory animals	Male athymic BALB/c nude mice (6 weeks old), Prox1f/f male mice (C57BL/6 background, 2 weeks old) and Alb-Cre; Prox1f/f male mice (C57BL/6 background, 2 weeks old) were prepared from the Shanghai Model Organisms Center (Shanghai, China), KrasG12D male mice (C57BL/6-Kras (LSL-G12D), 8 weeks old) and Lkb1L/L male mice (C57BL/6 background, 8 weeks old) were originally generously provided by T. Jacks (Koch Institute for Integrative Cancer Research, Cambridge, MA, USA) and R. Depinho (MD Anderson Cancer Center, Houston, TX, USA), respectively, and a gift from Prof. Ji Hongbin (Shanghai Institute of Biochemistry and Cell Biology, Center for Excellence in Molecular Cell Science, Chinese Academy of Sciences, Shanghai, China) for current study. All mice were raised in specific pathogen-free conditions. Mice were housed with a 12-hour light/dark schedule at 25±1°C and were fed an autoclaved chow diet (XieTong Biology, Cat#1010013) and water ad libitum.
Wild animals	No wild animals were used in this study.
Field-collected samples	No field-collected samples were used in this study.
Ethics oversight	All animal experiments were undertaken in accordance with relevant guidelines and regulations and were approved by the Institutional Animal Care and Use Committee at Ren Ji Hospital affiliated to School of Medicine of Shanghai Jiao Tong University.

Note that full information on the approval of the study protocol must also be provided in the manuscript.

Human research participants

Policy information about studies involving human research participants

Population characteristics	All surgically removed specimens were collected from patients who underwent radical hepatectomy (n=90) or lung cancer resection (n=90) from 2011-2015. The diagnosis of liver or lung cancer cancers or normal tissues was confirmed based on

	histological findings by independent pathologists. Liver cancer patients, aged 33-83, 74.4% of men and 25.6% of women. Lung cancer patients, aged 32-78, 52.2% of men and 47.8% of women. All data were gathered retrospectively, and the survival periods were defined as months after surgery.
Recruitment	Written informed consent was acquired from the patients and the patients' parties. Patients who match the above characteristic were approached and recruited into the studies without any additional bias. Written informed consent was acquired from the patients and the patients' parties.
Ethics oversight	This study was approved by the Ethics Committees of Ren Ji Hospital affiliated to School of Medicine of Shanghai Jiao Tong University,approval number RA-2020-250.

Note that full information on the approval of the study protocol must also be provided in the manuscript.

Flow Cytometry

Plots

Confirm that:

☒ The axis labels state the marker and fluorochrome used (e.g. CD4-FITC).

☒ The axis scales are clearly visible. Include numbers along axes only for bottom left plot of group (a 'group' is an analysis of identical markers).

☒ All plots are contour plots with outliers or pseudocolor plots.

☒ A numerical value for number of cells or percentage (with statistics) is provided.

Methodology

Sample preparation	For cell apoptosis assay, around 1×106 cells were suspended in 1×Binding Buffer and incubated with annexin V-FITC and propidium iodide (PI) at room temperature (25 °C) for 15 min.
Instrument	FACS Calibur2Lasers
Software	FlowJo
Cell population abundance	No sorting was performed
Gating strategy	Gating strategy is provided in the manuscript.

☒ Tick this box to confirm that a figure exemplifying the gating strategy is provided in the Supplementary Information.
